# Unveiling the conserved nature of *Heliconia* chloroplast genomes: insights from the assembly and analysis of four complete chloroplast genomes

**DOI:** 10.3389/fpls.2024.1535549

**Published:** 2025-01-16

**Authors:** Xin Cheng, Chengcheng Shi, Ting Yang, Qijin Ge, W. John Kress, Xin Liu

**Affiliations:** ^1^ College of Life Sciences, University of Chinese Academy of Sciences, Beijing, China; ^2^ BGI Research, Beijing, China; ^3^ Department of Botany, National Museum of Natural History, Smithsonian Institution, Washington, DC, United States

**Keywords:** *Zingiberales*, *Heliconiaceae*, *Heliconia*, chloroplast genome, genomic features

## Abstract

**Introduction:**

*Heliconia*, a genus within the Zingiberales order, is renowned for its diverse morphology, suggesting a rich genetic reservoir. However, genetic research on plants within the *Heliconiaceae* family has primarily focused on taxonomy and phylogenetics, with limited exploration into other genetic aspects, particularly the chloroplast genome. Given the significance of chloroplast genomes in evolutionary studies, a deeper understanding of their structure and diversity within Heliconia is essential.

**Methods:**

In this study, we sequenced and assembled the complete chloroplast genomes of four representative Heliconia species: *Heliconia bihai, Heliconia caribaea, Heliconia orthotricha*, and *Heliconia tortuosa*. The chloroplast genomes were analyzed for structure, gene content, and nucleotide diversity. We also performed comparative analysis with other species within the Zingiberales order to investigate structural and functional differences.

**Results:**

The assembled chloroplast genomes of the four Heliconia species exhibited a typical quadripartite structure and ranged in length from 161,680 bp to 161,913 bp. All genomes contained 86 protein-coding genes. Comparative analysis revealed that the chloroplast genome structures of the different Heliconia species were highly conserved, with minor variations. Notably, the chloroplast genome of Heliconia was slightly shorter than those of other Zingiberales species, primarily due to the reduced length of the inverted repeat region. In terms of nucleotide diversity, Heliconia species exhibited lower diversity in their chloroplast genomes compared to other families within the Zingiberales order.

**Discussion:**

This study provides valuable insights into the conserved nature of the chloroplast genome in Heliconia. The reduced chloroplast genome size, particularly the shortened inverted repeat region, marks a distinct feature of Heliconia within the Zingiberales family. Our findings also underscore the low nucleotide diversity within the chloroplast genomes of Heliconia species, which could be indicative of their evolutionary history and limited genetic differentiation. These results contribute to a broader understanding of chloroplast genome evolution in the Zingiberales and offer important genetic resources for future research on Heliconia and related species.

## Introduction

*Heliconia*, a genus belonging to the *Heliconiaceae* family, is a unique group of flowering plants comprising nearly 200 species (Iles et al., 2017; Linares et al., 2020). These plants are primarily found in tropical America and certain islands in the western Pacific. The inflorescence of Heliconia is a defining feature that makes it highly popular in horticulture, thanks to its vibrant, waxy bracts that attract pollinators. These bracts form part of an upright or pendulous cone-like structure, with the true flowers hidden within. A well-known previous study investigated the relationship between the beak characteristics of hummingbirds and the appearance of *Heliconia* bracts on two islands in the Lesser Antilles. The ecological structure of the islands further supports the coevolution between *Heliconia* and hummingbirds (Altshuler and Clark, 2003; Temeles and Kress, 2003). Additionally, the exotic bracts of Heliconia are in high demand in the global fresh-cut flower market (Linares et al., 2020), further highlighting their ecological and economic significance. The ecological significance of the genus in tropical forests, as well as its taxonomic and morphological aspects, have garnered considerable interest.

The high morphological diversity of Heliconia initially led taxonomists to classify the genus based on appearance (Kress, 1984; Lennart, 1992; Kress et al., 1999; Kress et al., 2001). Genetic markers from plastid and nuclear genomes were employed to study the evolution of Heliconia, revealing that its diversity originated in the Late Eocene (39 million years ago) and underwent rapid diversification during the Early Miocene. However, studies specifically addressing the molecular diversity of Heliconia remain limited, primarily using genetic markers to investigate evolution at the species and population levels (Marouelli et al., 2010; Suárez-Montes et al., 2011; Côrtes et al., 2013; Stein et al., 2014; Westerband and Horvitz, 2015). Previous studies utilized Amplified Fragment Length Polymorphism (AFLP) makers to study cultivated *Heliconia* species (Isaza et al., 2012) and the genetic diversity of *H. bihai* populations (Marouelli et al., 2010; Martén-Rodríguez et al., 2011; Suárez-Montes et al., 2011; Isaza et al., 2012; Côrtes et al., 2013; Stein et al., 2014; Westerband and Horvitz, 2015). Random Amplified Polymorphic DNA (RAPD) markers were also applied to study evolutionary relationship among *Heliconia* species, revealing the monophyletic nature of the *Heliconia* genus (Marouelli et al., 2010). Furthermore, In the larger group of Zingiberales order, to which *Heliconia* belongs, more genetic markers or representative whole chloroplast genomes were utilized to depict the evolutionary process of Zingiberales species, indicating *Heliconia* as the sister group to the remaining families in Zingiberales (Barrett et al., 2013; Barrett et al., 2014). A key factor that has hindered accurate phylogenetic reconstructions in many tropical plant groups is the widespread occurrence of rapid lineage radiations (Couvreur et al., 2014; Koenen et al., 2015). To gain a more comprehensive understanding of the correlation between morphological and molecular diversity in Heliconiaceae, it is crucial to gather more extensive molecular data. This includes focusing on genes that are sufficiently long to provide substantial phylogenetic signal while filtering out genes under strong selection (Lemmon and Lemmon, 2013). Comparative analyses with other species within the Zingiberales order will further contribute to elucidating the evolutionary patterns and relationships among Heliconia species, as well as their coevolutionary dynamics with hummingbirds.

In this study, we assembled the chloroplast genomes of four representative Heliconia species, including Heliconia bihai, Heliconia caribaea, Heliconia orthotricha, and Heliconia tortuosa (Pic. S1). We conducted a thorough examination of the complete chloroplast genome structures of these species, performing detailed analyses and comparisons of their structural and genomic features with those of other species in the Zingiberales order.

## Materials and methods

### Plant materials and DNA sequencing

Four representative *Heliconia* species, namely *Heliconia bihai* (L.) “Yellow Dancer”, *Heliconia caribaea* Lam., *Heliconia orthotricha*, and *Heliconia tortuosa* Griggs, were selected for our study. *H. bihai*, *H. caribaea*, and *H. tortuosa* samples were collected from Gardens of Plant Group Hawai’i by Kress lab, while *H. orthotricha* was obtained from the Guangdong Flower Market. Fresh leaves were carefully collected and immediately snap-frozen in liquid nitrogen. The samples were then stored at -80 °C until DNA extraction. DNA extraction was performed using the modified CTAB method (Aboul-Maaty and Oraby, 2019). Subsequently, the DNA samples were sequenced on BGISEQ-500 platforms (MGI, Shenzhen, China) using the whole genome strategy at BGI Research Qingdao lab, following the manufacturer instructions (Huang et al., 2017).

### Chloroplast genome assembly and annotation

The *de novo* assembly of four chloroplast genomes was performed using NOVOplasty (version 4.3.3) (Dierckxsens et al., 2016)with parameters of “Genome Range: 150,000-190,000; K-mer: 31; Seed Input: Heliconia collinsiana; Combined reads: All clean reads”. For the homology-based assembly of the chloroplast genomes, MITObim version 1.9.1 (relies on MIRA 4.0.2) (https://github.com/chrishah/MITObim) was utilized with parameters of “Read Pool: Extracted all clean reads with a depth of 20×; -quick Heliconia collinsiana” (Hahn et al., 2013). The resulting assemblies from both methods were then aligned and refined against the reference chloroplast genome of *Heliconia collinsiana* (NC_020362.1). Each assembled complete chloroplast genome underwent annotation utilizing GeSeq (Tillich et al., 2017)and the online CPGAVAS2 (an integrated Plastome Annotator and Analyzer) (Shi et al., 2019) with default parameters. Subsequently, the newly annotated chloroplast genome sequences were initially validated using the online tool GB2sequin (Lehwark and Greiner, 2019), further verified, and formatted using Sequin v. 15.50 from NCBI before being deposited in GenBank (accession numbers provided in **Table 1**).To visualize the chloroplast genome maps, the online program OGDRAW v1.3.1 (Greiner et al., 2019) (https://chlorobox.mpimp-golm.mpg.de/OGDraw.html) was employed.

**Table 1:** General characteristics of four *Heliconia* chloroplast genomes.

Charateristics and parameters	*Heliconia bihai*	*Heliconia caribaea*	*Heliconia orthotricha*	*Heliconia tuotorsa*
Total cp genome size (bp)	161,745	161,908	161,689	161,672
LSC length (bp)	89,772	89,861	89775	89,734
SSC length (bp)	18,757	18,779	18656	18,704
IR length (bp)	26,608	26,634	26629	26,617
Total number of genes	132	132	132	132
CDS genes	86	86	86	86
rRNAs genes	8	8	8	8
tRNAs genes	38	38	38	38
Total GC content (%)	37.36	37.34	37.36	37.36
GC content for LSC (%)	35.39	35.36	35.39	35.38
GC content for SSC (%)	31.29	31.27	31.29	31.34
GC content for IR (%)	42.82	42.83	42.82	42.82
Coding GC (%)	38.17	38.17	38.18	38.13
1st codon position GC (%)	45.74	45.75	45.82	45.68
2nd codon position GC (%)	38.41	38.41	38.47	38.40
3rd codon position GC (%)	30.37	30.35	30.24	30.30

### Chloroplast genome analysis and statistics

The identification of simple sequence repeats (SSRs) was performed using the online MISA-web tool (Thiel et al., 2003; Beier et al., 2017). The minimum number of repeats was set to 10, 5, 4, 3, 3, and 3 for mononucleotide (mono-), dinucleotide (din-), trinucleotide (tri-), tetranucleotide (tetra-), pentanucleotide (penta-), and hexanucleotide (hexan-) SSRs, respectively (Martin et al., 2013). Tandem repeat sequences were detected using Tandem Repeats Finder with default parameters (Benson, 1999). The parameters used were 2, 7, and 7 for weights of match, mismatch, and indels, respectively. The detection parameters were set to 80 for the matching probability (Pm), 10 for the indel probability (Pi), a minimum alignment score of 50, and a maximum period size of 500. Long repeat sequences were analyzed using REPuter (Kurtz, 2001). The analysis identified forward (F), reverse (R), complement (C), and palindromic (P) repeats with default parameters. The parameters used were, ‘-f’ to compute maximal forward repeats, ‘-p’ to compute maximal palindromes, ‘-h’ to search for repeats up to the given hamming distance, and ‘-l’ to specify the desired length of repeats. Codon usage was analyzed using MEGA11 (Kumar et al., 2008), and the relative synonymous codon usage (RSCU) and amino acid frequencies were calculated with default settings. Additionally, the GC content of the three positions was analyzed using CUSP in the EMBOSS program (Rice et al., 2000).

### Comparative analysis of the chloroplast genomes

DNA polymorphisms, identified by calculating nucleotide diversity (π) using DnaSP (DNA Sequence Polymorphism) v5.10.1 (Librado and Rozas, 2009), were used to detect highly variable sites among chloroplast genomes in different evolutionary clades. Alignments of reordered whole-chloroplast genome sequences, obtained using MAFFT v7.407 (Katoh and Standley, 2013), were sliced into 800-site windows to calculate nucleotide diversity with a step size of 200 sites. Sites with gaps and plastid sequences with rearrangements were excluded. Signals of natural selection were evaluated for all protein coding genes. The non-synonymous (Ka) and synonymous (Ks) substitution ratio (Ka/Ks) of each gene was calculated in the background of different species in Zingiberales. The protein sequences of protein coding genes in each pair of the species were aligned using MAFFT (v7.407) (Katoh and Standley, 2013). Subsequently, the coding DNA sequences (CDS) were converted into codon alignments based on the protein sequence alignment using the Perl script pal2nal (v14) (Suyama et al., 2006). The KaKs calculator (v2.0) (Wang et al., 2010), utilizing its model-averaging method, was employed to compute the values for Ka (non-synonymous substitutions), Ks (synonymous substitutions), and the Ka/Ks ratio.

The pairwise alignments and sequence divergence analysis were conducted for *H. bihai*, *H. caribaea*, *H. orthotricha*, and *H. tortuosa*, along with seven additional Zingiberales species, namely *Canna indica* (MK561603), *Costus pulverulentus* (KF601573), *Musa acuminata* (NC_058940), *Orchidantha fimbriata* (KF601569.1), *Thaumatococcus daniellii* (KF601575.1), *Ravenala madagascariensis* (NC_022927.1), and *Zingiber officinale* (NC_044775). The alignments and sequence comparisons were performed using the mVISTA tool with LAGAN and Shuffle-LAGAN modes (Brudno et al., 2003). The analysis was carried out to assess the contraction and extension of the inverted repeat (IR) borders across the four major regions (LSC/IRa/SSC/IRb) in the chloroplast genome sequences of all eleven species. This assessment was carried out using the web tool IRscope (Amiryousefi et al., 2018).

### Phylogenetic analysis

We obtained 22 chloroplast genomes from the NCBI database. In addition to the seven species from the Heliconiaceae family, we included 45 additional species in our analysis and used the monocotyledonous plant rice (*Oryza sativa*) as an outgroup. Subsequently, we utilized the HomBlocks pipeline (Bi et al., 2018) to efficiently identify homologous blocks among organelle genomes and extract phylogeny-informative regions for constructing a multi-gene alignment. This method leverages core conserved fragments, including coding genes, functional non-coding regions, and rRNA, to generate high-quality and informative data matrices.

Maximum likelihood (ML) analysis was performed using the IQ-TREE program (Minh et al., 2020)with the parameter ‘-m GTR+G+I -bb 1000 -bnni -cmax 15’ as the nucleotide substitution model (Li et al., 2023). MEGA11 was used with default parameters to construct the Neighbor-Joining evolutionary tree. To visualize the phylogenetic relationships, we utilized the iTOL online tool (https://itol.embl.de/) (Letunic and Bork, 2021).

For the analysis of shared genes among the 52 species, we generated a high-quality alignment file using the MAFFT (Katoh and Standley, 2013) with default parameters. These alignment files, along with the chloroplast genome sequences, were used as input files for codeml. In the initial run, the ctl file parameters were set to ‘runmode = 0, CodonFreq = 2, and model = 0’. In the second run, the parameters were adjusted to ‘mode = 2’, focusing on the *Heliconia*ceae family as the foreground branch, allowing for the calculation of different evolutionary rates (Librado and Rozas, 2009). The DnaSP v5 software (Librado and Rozas, 2009) was employed to compare the aligned sequences, calculate nucleic acid diversity, and obtain the value of π.

## Results

### Assembly of *Heliconia* chloroplast genomes

**Figure 1:**
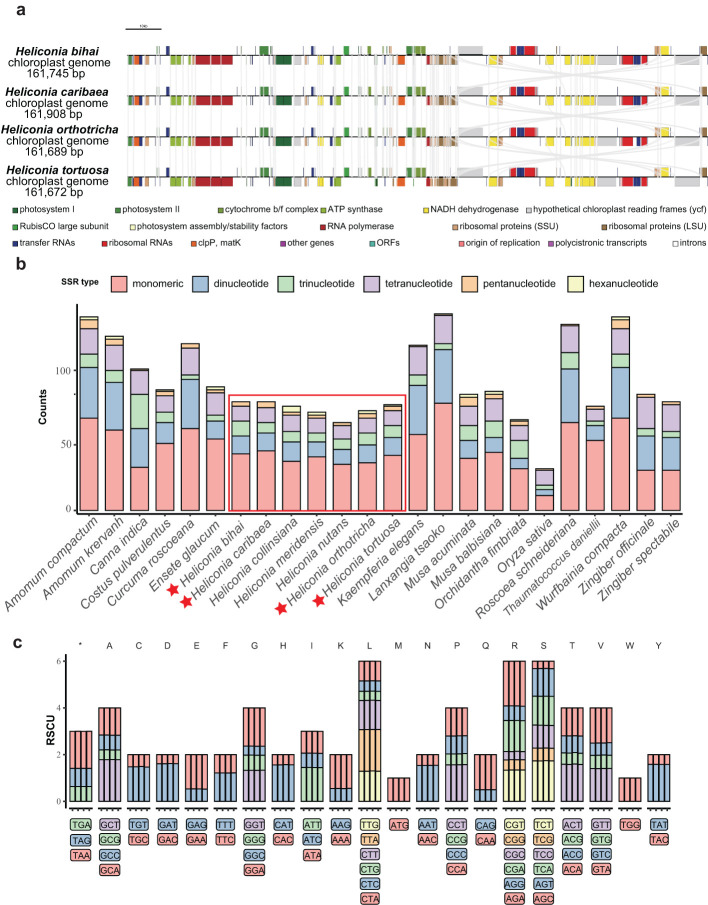
Chloroplast genomes of four *Heliconia* species. **(A)** Genetic features. Genes were shown along the four chloroplast genomes shown in linear forms. **(B)** Simple sequence repeats (SSRs) compositions. Types and numbers of SSRs in the four chloroplast genomes comparing to those of related species. **(C)** Codon usage patterns. Codon usage patterns of the four chloroplast genomes are shown in order.

Utilizing the sequencing data, the chloroplast genomes of four Heliconia species (*H. bihai*, *H. caribaea*, *H. orthotricha*, and *H. tortuosa*) were assembled (**Supplementary Table S1**). It was found that the chloroplast genomes of these Heliconia species exhibit a high degree of similarity. The sizes of the chloroplast genomes were as follows: 161,745 bp for *H. bihai*, 161,908 bp for *H. caribaea*, 161,689 bp for *H. orthotricha*, and 161,672 bp for *H. tortuosa*. A total of 132 genes were identified in these chloroplast genomes, comprising 86 coding sequences (CDS), 8 ribosomal RNAs (rRNAs), and 38 transfer RNAs (tRNAs) (**Figure 1A**; **Table 1**; **Supplementary Table S2**). Of these genes, 18 were identified as intron-containing genes in *H. bihai, H. orthotricha*, and *H. tortuosa*, with 16 of them containing a single intron each, while two genes (*clpP* and *ycf3*) had two introns each. It is noteworthy that *H. caribaea* possesses 16 splitting genes in addition to trna, a feature that differentiates it from the other three species with regard to the number of splitting genes (see **Supplementary Table S3**). The chloroplast genomes of these four Heliconia species exhibit a quadripartite structure, a characteristic shared by the majority of angiosperms. This structure consists of a large single-copy (LSC) region (89,772 bp for *H. bihai*, 89,861 bp for *H. caribaea*, 89,734 bp for *H. orthotricha*, and 89,775 bp for *H. tortuosa* two inverted repeat (IR) regions (26,608 bp for *H. bihai*, 26,634 bp for *H. caribaea*, 26,617 bp for *H. orthotricha*, and 26,629 bp for *H. tortuosa*) (**Supplementary Figure S1**). The GC content in the LSC, SSC, and IR regions of all four chloroplast genomes was found to be 35.4%, 31.3%, and 42.8%, respectively (**Table 1**). The higher GC content observed in the IR regions may be attributed to the abundance of rRNA and tRNA genes, which inherently have a relatively higher GC content.

### Features of *Heliconia* chloroplast repeat sequence

Our analysis reveals that while the repetitive sequences in the chloroplasts of various *Heliconia* species display quantitative similarities, they differ in their types. Focusing on SSRs, we found minimal variation in their numbers among the four *Heliconia* genomes, with 73 in *H. bihai* and *H. caribaea*, 71 in *H. tortuosa*, and 68 in *H. orthotricha*. However, despite the similarity in the number of encoded genes, notable differences in SSR types were observed. Specifically, *H. bihai* and *H. caribaea* featured monomeric, dinucleotide, trinucleotide, tetranucleotide, and pentanucleotide SSR types, whereas *H. tortuosa* and *H. orthotricha* additionally included the hexanucleotide SSR type in the SSC region (**Supplementary Table S4**). Most SSRs were concentrated in the LSC regions, with only one SSR located within coding genes across all four *Heliconia* species.

When comparing the chloroplast genome data of other sequenced species within the *Zingiberales* order (**Figure 1B**), we observed that the presence of both ACT and AATC types of SSRs in the genome could potentially serve as an indicator for classifying a species as belonging to the *Heliconia* genus (**Supplementary Table S5**).

Turning to tandem repeats (TRs), our detailed analysis revealed that most repeat units were predominantly composed of A or T, with the longest repeat sequence spanning approximately 120 base pairs (**Supplementary Table S6**). Shifting to dispersed repeats (DRs), *H. bihai* exhibited two types (forward repeat and reverse repeat), while *H. caribaea* and *H. tortuosa* showed three types (forward repeat, reverse repeat, and palindromic repeat). In contrast, *H. orthotricha* possessed all four types of dispersed repeats (forward repeat, reverse repeat, complemented repeat, and palindromic repeat), though it had a comparatively lower quantity of DRs.

Overall, these findings highlight the distinctive repeat features in *Heliconia* chloroplasts, which could serve as valuable genetic markers for distinguishing *Heliconia* species from one another and from other species.

### Features of *Heliconia* chloroplast coding genes

Beyond repeat features, we further explored the protein-coding genes within the chloroplast genome to uncover potential factors linked to the visual diversity of *Heliconia* and its successful proliferation in tropical forest ecosystems. Codon usage bias refers to the uneven utilization of different codons that encode the same amino acid within a genome. In our analysis of the 86 CDS in chloroplast genomes, we computed the frequency of codon usage and relative synonymous codon usage (RSCU) (**Figure 1C**; **Supplementary Table S7**). The CDS in these chloroplast genomes encode 20 amino acids using 64 codons, including the termination codon. Among these 64 codons, 30 of them exhibit an RSCU value greater than 1, with 29 of them ending with an A or T bases. This observation indicates a preference for A or T endings in the codons of the *Heliconia* chloroplast genomes, which is consistent with the previously mentioned decrease in GC content at the third position of codons (30.3%) compared to the first (45.7%) and second (37.4%) positions. Regarding the codon usage bias among the four chloroplast genomes, there are six codons each for arginine (Arg), leucine (Leu), and serine (Ser), while only one codon each is present for methionine (Met) and tryptophan (Trp). Within the spectrum of amino acids, Isoleucine (Ile) stands out as the most frequently occurring amino acid, predominantly encoded by the ATT codon with a frequency of 41%. Conversely, cysteine (Cys) is the least common amino acid, with the TGC codon having the lowest frequency at 3%, across four chloroplast genomes. Except for methionine (Met) and tryptophan (Trp), nearly all amino acids are encoded by 2–6 synonymous codons.

Selective pressure analysis provides insights into the chloroplast genes under selection and nucleotide diversity within specific genes in the chloroplast genomes. During the positive selection analysis of the genes used in constructing the phylogenetic tree, we observed that *Heliconia*, as a foreground branch, did not undergo significant positive selection. However, within the *Heliconiaceae* family, three genes (*ndhD*, *rpl2*, and *ycf2*) showed a trend of positive selection (Ka/Ks > 1) (**Supplementary Table S8**).

**Figure 2:**
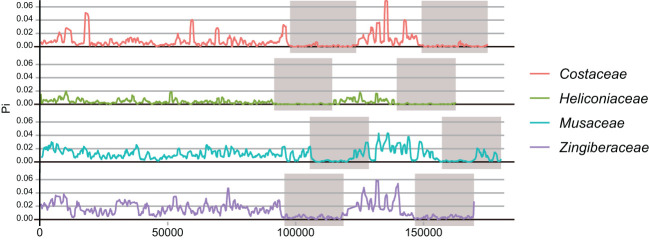
Nucleotide diversity of phylogenetic clades containing Zingiberales species. The curved line depicts the fluctuation of ╥ values across the genome alignment. The shadow layers in grey indicate the approximate range of IRs regions.

The nucleotide diversity (Pi) of complete chloroplast genomes was analyzed separately for four families within *Zingiberales*: *Costaceae*, *Heliconiaceae*, *Musaceae*, and *Zingiberaceae* (**Figure 2**). The chloroplast genomes of Heliconiaceae plants exhibit lower nucleotide diversity (π) across the entire genome compared to other evolutionary lineages. Additionally, there is reduced variation in diversity across different genomic regions, as evidenced by the smaller difference in π values between the inverted repeat (IR) and single-copy (SC) regions in Heliconiaceae compared to other plant groups. Additionally, focusing on protein-coding genes, we analyzed nucleotide diversity in a total of 12 species from the Zingiberales order (**Supplementary Table S9**). Among these genes, the *ndhD* gene exhibited notably high nucleotide diversity, with a PAI (per-site average information) value exceeding 0.2. Several other genes, including *ccsA*, *cemA*, *infA*, *matK*, *ndhD*, *rpl*, *rpo*, *rps* also displayed PAI values greater than 0.05. However, among the *Heliconia* species, we did not observe coding genes with high nucleotide diversity (**Supplementary Table S10**).

### Structural comparison within *Zingiberales* chloroplast genomes

**Figure 3:**
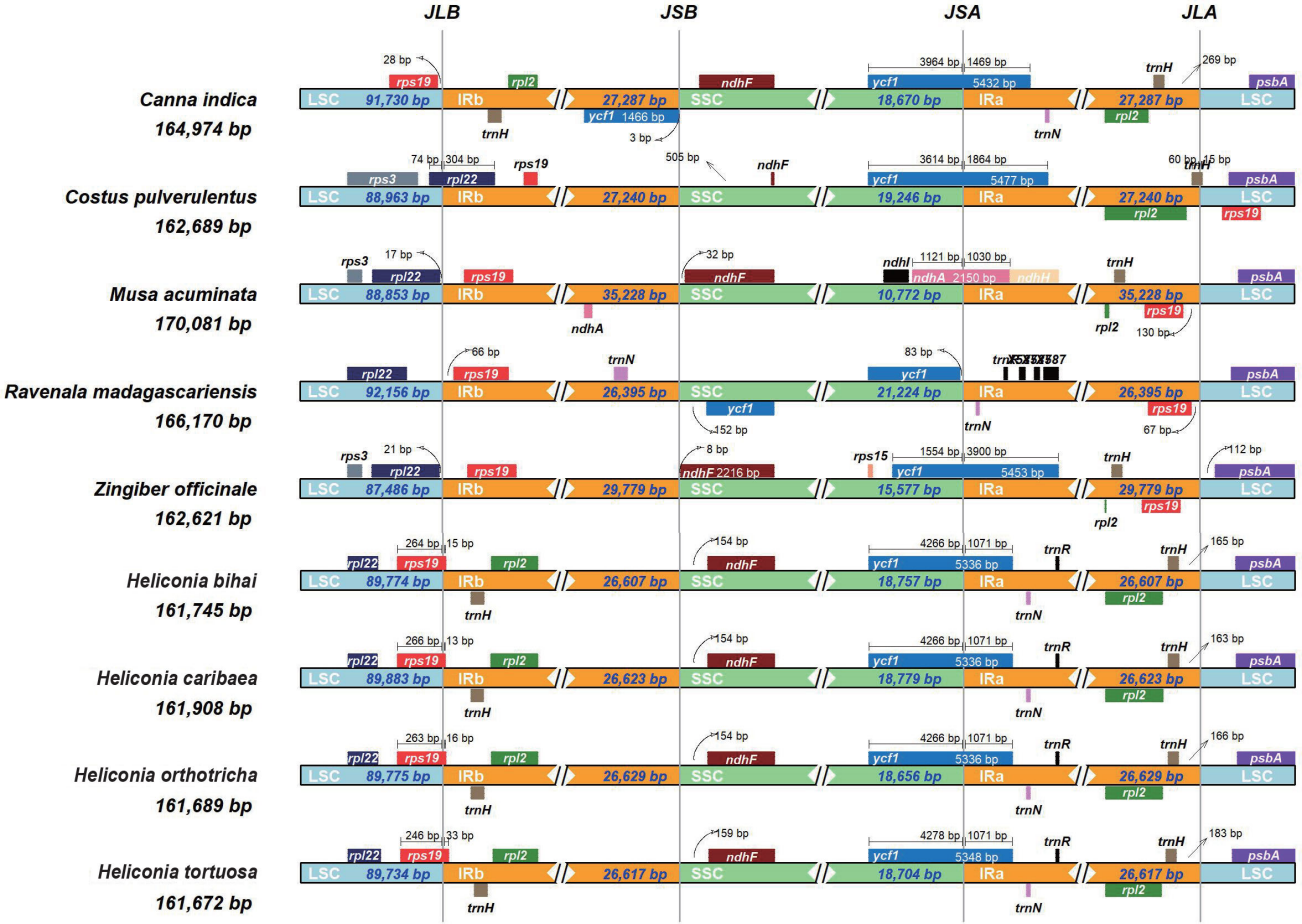
Structural variations in the chloroplast genomes. Chloroplast genomes of eight Zingiberales species are compared to indicate the major chloroplast genome regions including LSC, SSC and IR regions. Genes transcribed forward are shown above the lines, whereas genes transcribed reversely are shown below the lines. Gene lengths in the corresponding regions are displayed above the boxes of gene names. JLB (LSC/IRb), JSB (IRb/SSC), JSA (SSC/IRa) and JLA (lra/LSC) denoted the junction sites between each corresponding two regions.

In our comparative analysis of *Heliconia* chloroplast genomes alongside five closely related species (*Canna indica*, *Costus pulverulentus*, *Musa acuminata*, *Ravenala madagascariensis*, *Zingiber officinale*), we observed remarkable structural conservation in the overall of the chloroplasts among species within the Zingiberales order. Specific structural variations were identified at distinct boundaries, including LSC/IRb, IRb/SSC, SSC/IRa, and IRa/LSC (**Figure 3**). These boundary regions in the four *Heliconia* species remained consistent yet exhibited unique features, setting them apart from other plants in *Zingiberale*. Noteworthy is the absence of the *rps19* gene in the IR region of *Heliconia* chloroplasts, distinguishing it from other *Zingiberales* plants where the IR region includes the rps19 gene. Furthermore, an elongated separation of approximately 150 base pairs at the boundary between the inverted repeat B (IRb) and the small single-copy region (SSC) in the *Heliconia* chloroplast genomes for the *ndhF* gene was noted. This contrasts with other species, where the distance typically falls within the range of approximately 10 to 60 base pairs. contraction in the inverted repeat (IR) region resulted in a slightly smaller chloroplast genome size in Heliconia compared to other species in the Zingiberales order.

**Figure 4:**
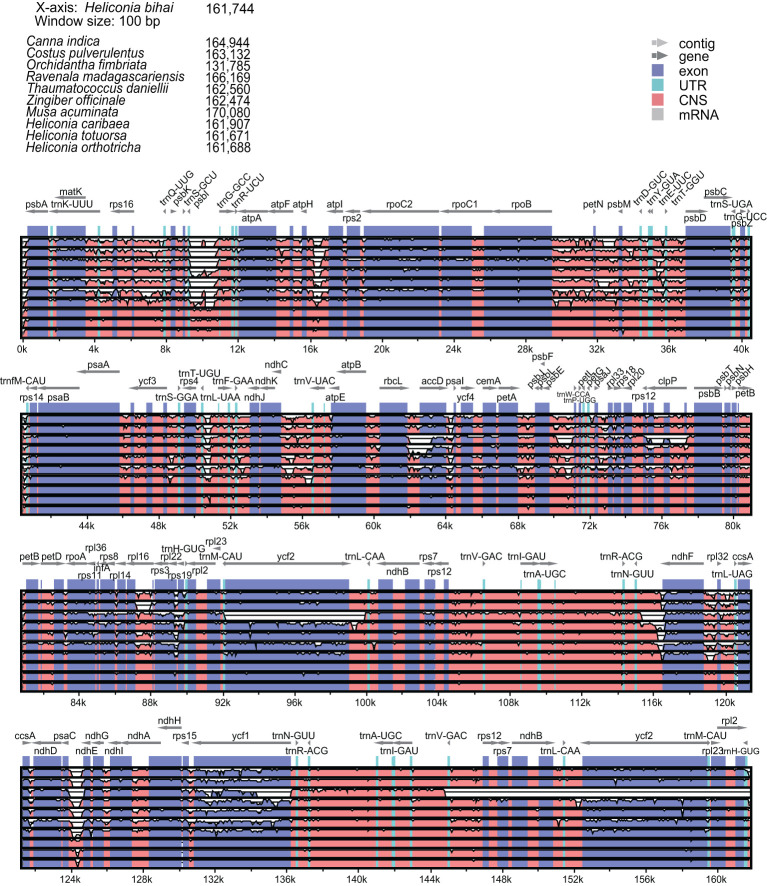
Comparing the four *Heliconia* chloroplast genomes to these of the other Zingiberales species. Chloroplast genomes are shown with genes indicated, and the vertical scale indicates the percentage of identity, ranging from 50% to 100%.

We conducted a sequence comparative analysis of *Heliconia* chloroplast genomes and those of related species. Using ***H. bihai*** as the reference, we compared its chloroplast genome sequence with those of *Canna indica*, *Costus pulverulentus*, *Musa acuminata*, *Orchidantha fimbriata*, *Thaumatococcus daniellii*, *Ravenala madagascariensis*, and *Zingiber officinale* from the *Zingiberales* order (**Figure 4**). The analysis revealed significant genetic diversity and variation in Heliconia compared to other Zingiberales plants, particularly in the conserved noncoding sequences (CNS), especially within the LSC and SSC regions. A similar analysis within the *Costaceae* and *Musaceae* families further confirmed the extensive conservation of chloroplast genomic sequences in *Heliconiaceae*. Based on the currently available data and considering the incomplete genomic data for other families within the Zingiberales order, *Heliconiaceae* species emerged as having the most conserved chloroplast genomes.

### Phylogeny of *Heliconia* species revealed by chloroplast genomes

**Figure 5:**
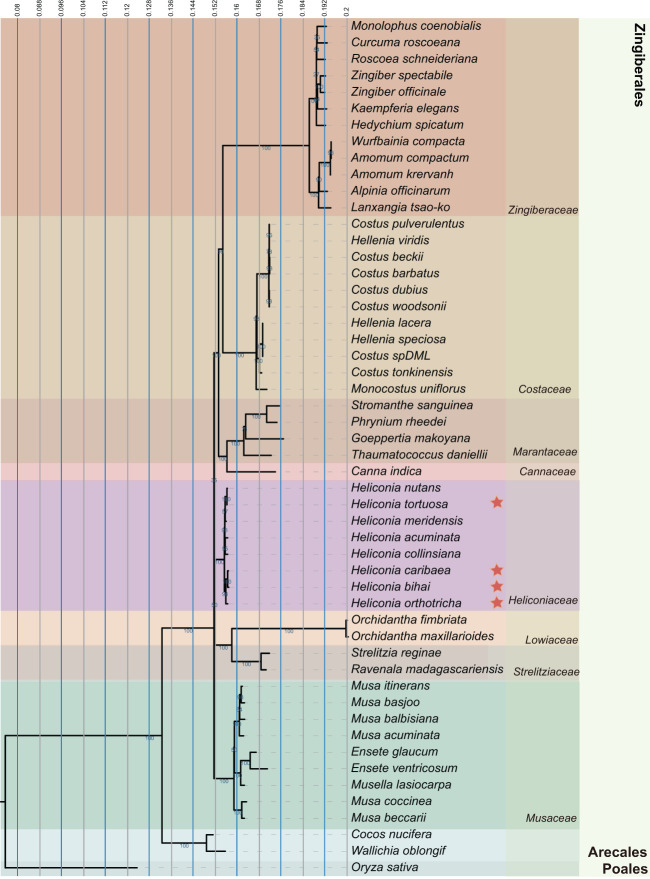
Phylogenetic tree of *Heliconia* and related species. Maximum likelihood (ML) phylogenetic tree was constructed for 52 species from Zingiberales order, and rice (*Oryza sativa)* as an outgroup. The confidence level of the phylogenetic tree are shown for each branch.

Analyzing the complete chloroplast genome yields more reliable results, providing substantial insights into the genetic evolution of plant species. We carefully selected 51 diverse plant species, representing major clades of Zingiberales plants (**Figure 5**), and including representatives from different families such as *Cannaceae*, *Costaceae*, and *Heliconia*ceae, *Musaceae*, *Marantaceae*, *Lowiaceae*, along with *Strelitziaceae* and *Zingiberaceae*. To construct multi-gene alignments, homologous blocks were identified among the organelle genomes, allowing for the efficient extraction of phylogeny-informative regions. By integrating core conserved fragments—comprising coding genes, functional non-coding regions, and rRNA—into a unified sequence for each genome, we minimized the impact of incomplete chloroplast genomes on the accuracy of phylogenetic tree construction. Phylogenetic trees were constructed using two methods: maximum likelihood (ML) and neighbor-joining (NJ). In the maximum likelihood (ML) tree, Zingiberales diverge from three distinct terminal nodes. *Heliconia*ceae plants formed a distinct branch, and emerge as the sister clade to *Musaceae*, *Strelitziaceae*, and *Lowiaceae*. While in the Neighbor-Joining tree, Zingiberales diverge from two distinct terminal nodes. Musaceae emerged as sister branches to *Heliconia*ceae and *Strelitziaceae*, forming a distinct clade (**Supplementary Figure S5**).

## Discussion

The high conservation of chloroplast genomes in terrestrial plants encompasses their structure, length, and gene content. In our study, we successfully assembled complete chloroplast genomes of Heliconia plants, closely resembling the reported structure of *Heliconia collinsiana*. Three main types of repeat sequences were found in organelle genomes, including simple sequence repeats (SSRs) (Song et al., 2014), tandem repeats (TRs), and dispersed repeats (DRs). Among these, SSRs exhibited high variability within a species, making them valuable markers for population genetics and phylogenetic analyses (Fan and Chu, 2007). Analysis of repetitive sequences, specifically SSRs, revealed distinguishable patterns not only among different Heliconia species but also across genera within the Zingiberales order.

Research on codon usage bias contributes to our understanding of genome evolution, gene expression regulation, and the adaptability of organisms to environmental changes (Parvathy et al., 2022). Our analysis reveals a codon usage bias favoring A or T endings in the codons of *Heliconia* chloroplast genomes. Additionally, the low GC content observed in both codon positions and repetitive sequences suggests a strong preference for A/T bases in Heliconia. highlighting the significance of studying codon usage patterns in understanding genome evolution and gene expression regulation.

To further explore functional sequence variations within highly conserved and maternally inherited chloroplast genomes, which can serve as valuable genetic markers for species differentiation (Wysocki et al., 2015). Compared to other Zingiberales species, the chloroplast genome of Heliconia is slightly shorter, which is attributed to a reduction in the length of the IR region. The relatively low nucleotide diversity in the chloroplast genomes of Heliconia indicates that the diverse appearances of these plants are not strongly correlated with variations in their chloroplast genomes.

The three genes showing a trend of positive selection include *ndhD*, *ycf2* and *rpl2*. Among them, *ndhD* exhibits significant nucleotide diversity across species in the Zingiberales order, particularly within the Zingiberales family, and is under positive selection. As a component of the chloroplast NADH dehydrogenase-like (NDH) complex, *ndhD* plays a crucial role in photosynthesis, particularly in electron transport interactions with photosystem I (PSI) (Peng et al., 2011; Shen et al., 2022). In addition, ***ycf2*** forms a complex with five nuclear-encoded FtsH-like proteins, known as the Ycf2-FtsHi complex. This complex functions as the import motor in land plants, facilitating the import of proteins into the chloroplast. Although its evolutionary conservation and functional specialization across photosynthetic organisms are well recognized, the mechanisms and broader evolutionary dynamics of this complex remain largely unexplored (Liang et al., 2024). On the other hand, the function of the *rpl2* gene remains unclear and requires further investigation.

Through comparative analysis of the chloroplast genomes of species within the Zingiberales order, certain genes exhibit significant nucleotide diversity, suggesting they may have evolved in response to diverse environmental conditions, contributing to the varied appearances observed among Zingiberales species. Genes such as *ccsA*, *cemA*, *infA*, *matK*, *ndhD*, *rpl*, *rpo*, *rps* encode proteins involved in various biological processes. For instance, ccsA encodes a crucial component in the synthesis of cytochrome c within the chloroplast (Xie and Merchant, 1996). *cemA* encodes a subunit of chloroplast ATP synthase involved in energy production during photosynthesis (Sonoda et al., 1999), whereas infA encodes a protein crucial for tRNA processing, contributing to chloroplast protein synthesis (Millen et al., 2001). Gene *matK* encodes a splicing enzyme that facilitates RNA splicing (Barthet and Hilu, 2007), These genes are vital for plant growth, development, and metabolic processes, supporting chloroplast structure and function. The findings of this study are consistent with those of previous investigations (Jiang et al., 2023; Li et al., 2023), reinforcing the notion that these genes have undergone adaptive evolution in response to environmental factors, further supporting their pivotal role in the molecular and functional diversity of species within the Zingiberales order.

Like the role of mitochondrial genomes in vertebrate genetics, chloroplast genomes have become a widely adopted tool for addressing phylogenetic and evolutionary questions. Chloroplast genomes, characterized by their maternal inheritance and relatively low mutation rates, are invaluable for elucidating phylogenetic relationships among green plants (Daniell et al., 2016). In our study, the chloroplast genome data have provided valuable insights into the evolutionary relationships within Zingiberales, highlighting the importance of methodological approaches in shaping the interpretation of these relationships. However, different methods for constructing phylogenetic trees may yield divergent results, and as such, determining the exact position of the Heliconia genus within the Zingiberales evolutionary tree remains challenging using chloroplast data alone. While current chloroplast genome resources offer critical genetic information for understanding the morphological diversity of Heliconia species, further research focused on complete nuclear genomes will be essential for a more comprehensive understanding of the genetic mechanisms underlying this diversity. The chloroplast genomes assembled in this study provide a solid foundation for such future investigations. Moreover, a deeper exploration of nuclear genome-encoded genes, particularly those related to gene retention and evolutionary processes, will be crucial in unveiling the evolutionary trajectory and functional diversity of *Heliconiaceae*.

## Conclusions

The analysis of *Heliconia* chloroplast genome repetitive sequences, specifically SSRs, revealed distinguishable patterns across genera within the Zingiberales order. Compared to other Zingiberales species, the chloroplast genome of Heliconia is slightly shorter, attributed to a reduction in the length of the IR region and an expansion at the SSC region boundary. The relatively low nucleotide diversity in the *Heliconia* chloroplast genomes suggests that the diverse appearances of Heliconias are not strongly correlated with chloroplast genome. Overall, comparative analysis from various perspectives indicates that the *Heliconia* chloroplast genomes are conserved within the *Heliconiaceae* family, while also displaying distinct characteristics that differentiate them from other species within the *Zingiberales* order.

## Data availability statement

The complete chloroplast genomes generated during the current study were deposited in NCBI database (PP093761, PP093760, PP093759, PP093762) and CNGB database (CNP0005095) . The other accession numbers for the remaining datasets analyzed in this study are listed in the **Table S11b**.
