# Supplementary material for: Unveiling the conserved nature of Heliconia chloroplast genomes: insights from the assembly and analysis of four complete chloroplast genomes
Source: Front Plant Sci. 2025 Jan 16;15:1535549. doi: 10.3389/fpls.2024.1535549 (PMC11779715; doi:10.3389/fpls.2024.1535549)
Supplement: Supplementary file 1 [file DataSheet1.zip › Supplementary_fig1.pdf]

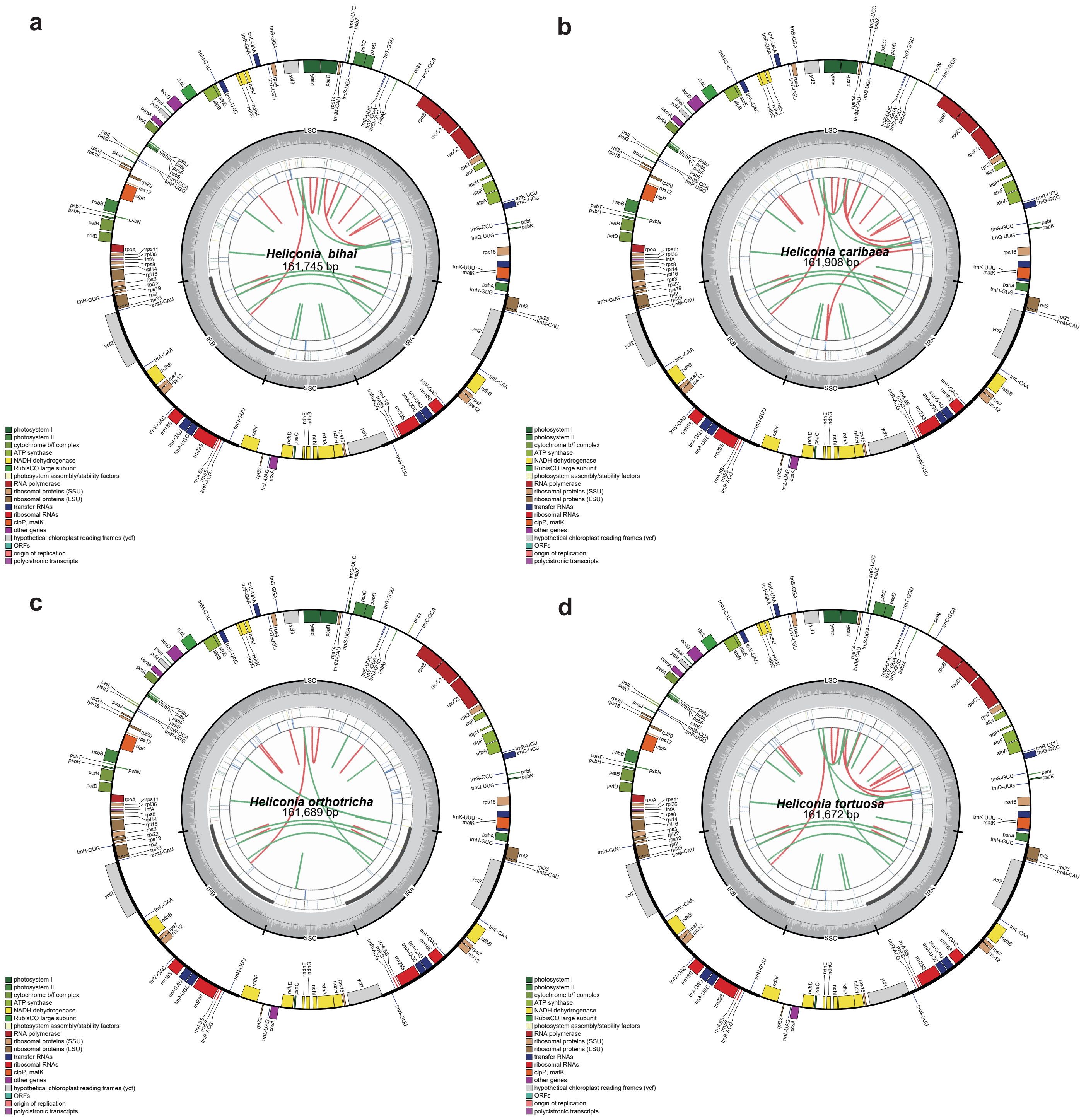

Supplementary Figure 1 | Gene map of the four *Heliconia* chloroplast genomes. Genes drawn outside the circle are counterclockwise, whereas inside are transcribed clockwise. Genes are color-coded according to different functional groups. The chloroplast genome is segmented into distinct regions, including Small Single Copy (SSC), Large Single Copy (LSC), and Inverted Repeats (IRa, IRb), each delineated for detailed analysis.The darker gray represents GC content in the inner circle, conversely the lighter one represents AT content. The centralmost illustration represents the genomic collinearity of the chloroplast genome. **a.** *Heliconia bihai* chloroplast genome, **b**. *Heliconia caribaea* chloroplast genome, **c**. *Heliconia orthotricha* chloroplast genome, **d**. *Heliconia tortuosa* chloroplast genome.
