# Supplementary material for: Unveiling the conserved nature of Heliconia chloroplast genomes: insights from the assembly and analysis of four complete chloroplast genomes
Source: Front Plant Sci. 2025 Jan 16;15:1535549. doi: 10.3389/fpls.2024.1535549 (PMC11779715; doi:10.3389/fpls.2024.1535549)
Supplement: Supplementary file 1 [file DataSheet1.zip › Supplementary_fig4.pdf]

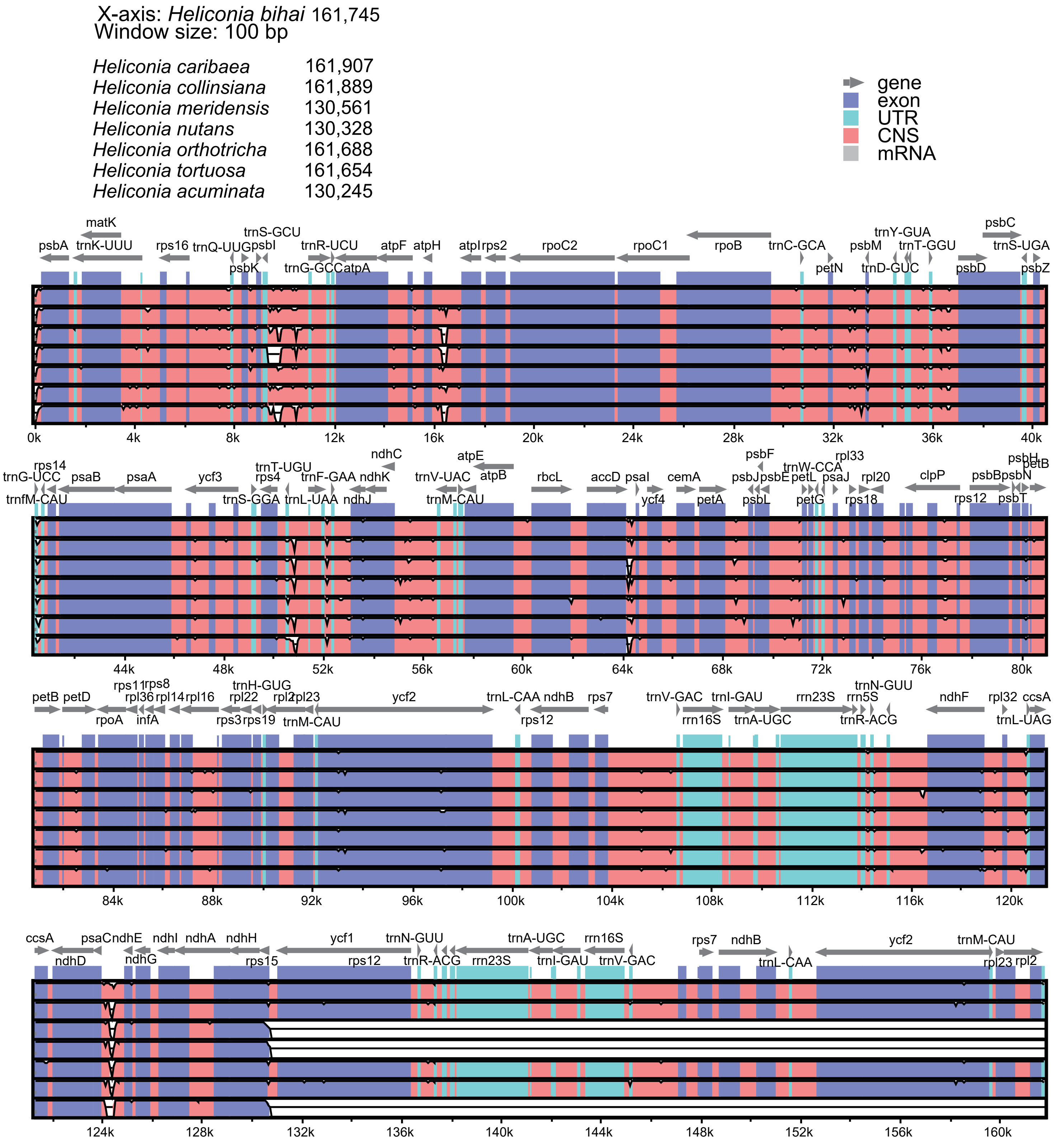

Supplementary Figure 4 | Comparing the *Heliconiaceae* chloroplast genomes
Chloroplast genomes are shown with genes indicated, and the vertical scale indicates the percentage of identity, ranging from 50% to 100%.
