# Supplementary material for: Unveiling the conserved nature of Heliconia chloroplast genomes: insights from the assembly and analysis of four complete chloroplast genomes
Source: Front Plant Sci. 2025 Jan 16;15:1535549. doi: 10.3389/fpls.2024.1535549 (PMC11779715; doi:10.3389/fpls.2024.1535549)
Supplement: Supplementary file 1 [file DataSheet1.zip › Supplementary_fig5.pdf]

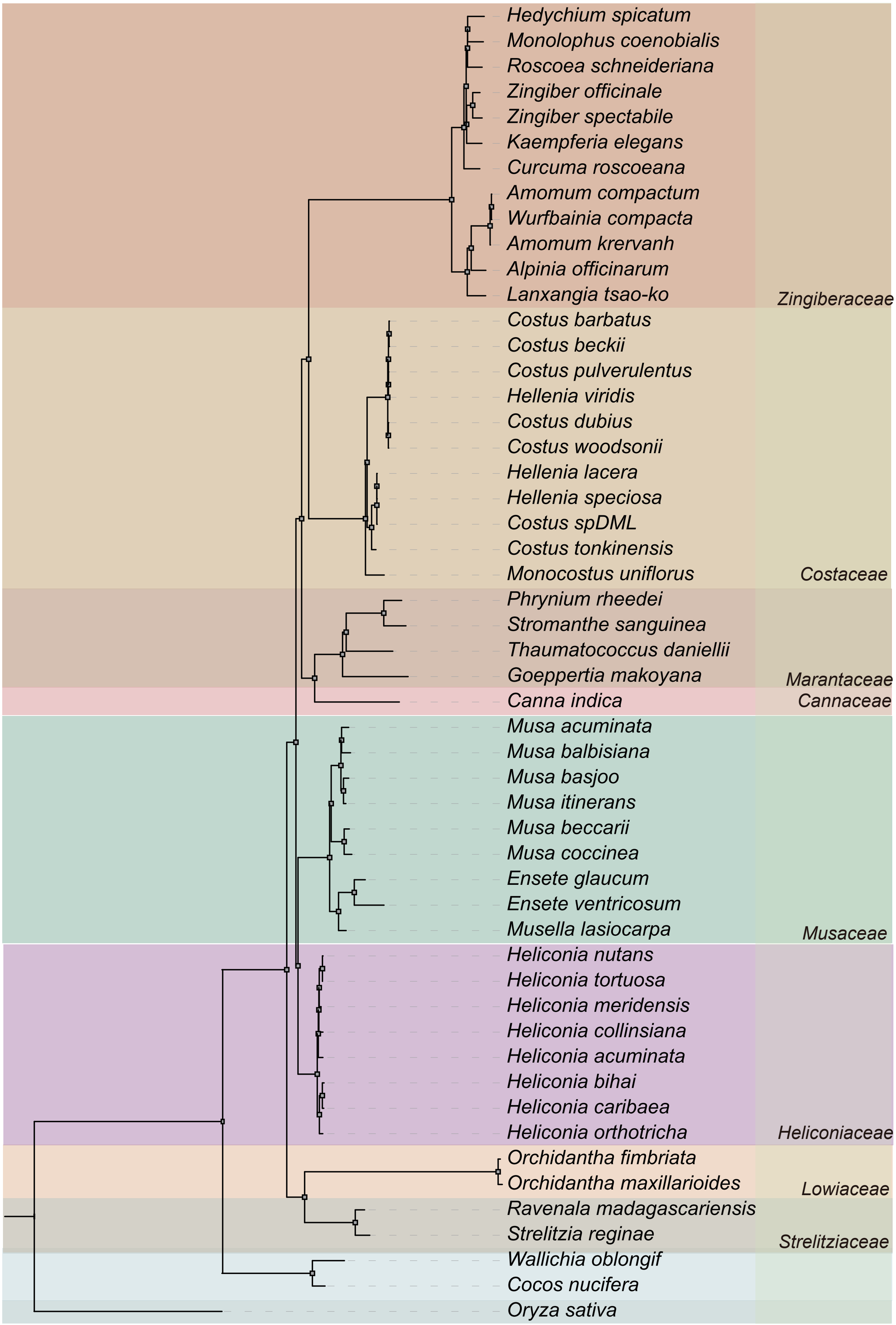

Figure S5 | Phylogenetic tree of Heliconiaceae and related species. Neighbor-joining (NJ) phylogenetic tree was constructed for 51 species from Zingiberales order, and rice (Oryza sativa) as outgroup.
