# Supplementary material for: Unveiling the conserved nature of Heliconia chloroplast genomes: insights from the assembly and analysis of four complete chloroplast genomes
Source: Front Plant Sci. 2025 Jan 16;15:1535549. doi: 10.3389/fpls.2024.1535549 (PMC11779715; doi:10.3389/fpls.2024.1535549)
Supplement: Supplementary file 1 [file DataSheet1.zip › Supplementary_pic1.pdf]

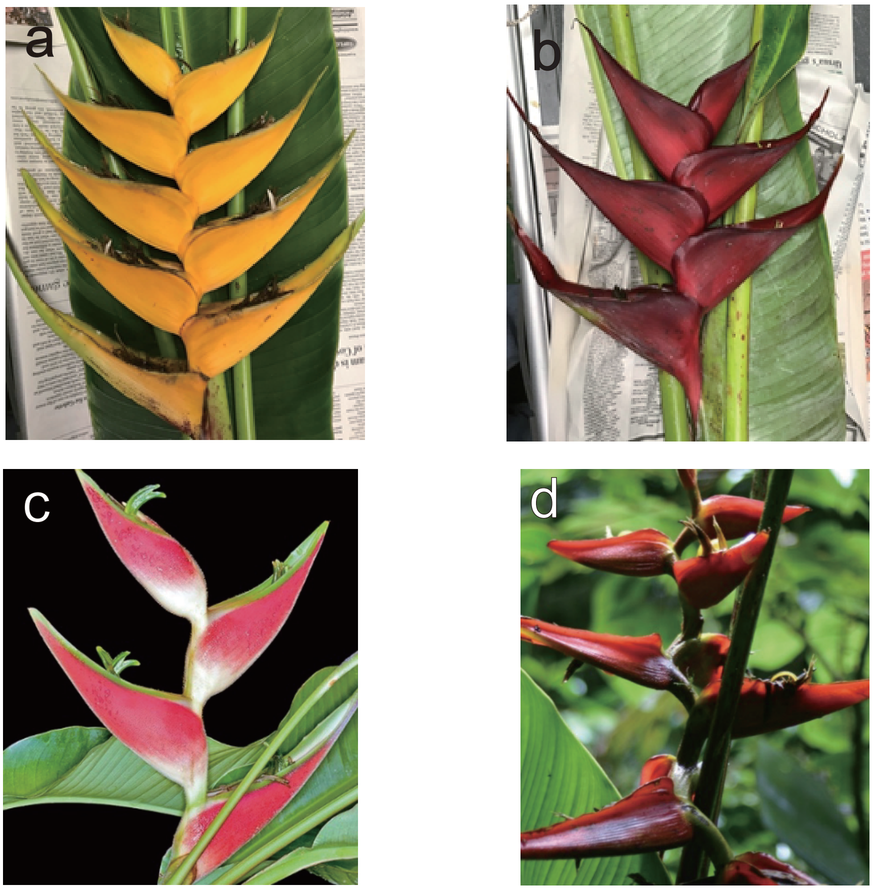

Picture S1 | Appearance of *Heliconiaceae*.
a. *Heliconia bihai* (L.) L. Cv. "Yellow Dancer." b. *Heliconia caribaea* Lam. Cv. "Black Magic."
c. *Heliconia orthotricha*. d. *Heliconia tortuosa*.
Source: "Heliconia tortuosa," Wikimedia Commons,
https://upload.wikimedia.org/wikipedia/commons/6/6e/Heliconia-tortuosa.jpg,
No machine-readable author provided. Codiferous assumed (based on copyright claims),
CC BY-SA 3.0, via Wikimedia Commons.
